# Fructose Stimulated Colonic Arginine and Proline Metabolism Dysbiosis, Altered Microbiota and Aggravated Intestinal Barrier Dysfunction in DSS-Induced Colitis Rats

**DOI:** 10.3390/nu15030782

**Published:** 2023-02-03

**Authors:** Ge Song, Qianyun Gan, Wentao Qi, Yong Wang, Meihong Xu, Yong Li

**Affiliations:** 1 Department of Nutrition and Food Hygiene, School of Public Health, Peking University, Beijing 100191, China; 2 Academy of National Food and Strategic Reserves Administration, Beijing 100037, China; 3 School of Health Science and Engineering, University of Shanghai for Science and Technology, Shanghai 200093, China

**Keywords:** fructose, arginine and proline metabolism dysbiosis, metabolome

## Abstract

The dysbiosis of intestinal microbiota and their metabolites is linked to the occurrence and development of metabolic syndrome. Although fructose has been proven to be associated with worsened mucus in the colon, its mechanism remains unclear. In this study, we evaluated the relatively low intake of sucrose and fructose in the experimental colitis of Sprague Dawley rats by investigating the microbiome and metabolome. Results showed that sucrose and fructose significantly reduced body weight, colon length and increased inflammation infiltration in colon. Sucrose and fructose worsen colon functions by inhibiting the expression of tight junction (TJ) protein ZO-1 and increasing the level of lipopolysaccharide neoandrographolide (LPS) in plasma, while fructose was more significant. Furthermore, sucrose and fructose significantly changed the composition of gut microbiota characterized by decreasing Adlercreutzia, Leuconostoc, Lactococcus and Oscillospira and increasing Allobaculum and Holdemania along with reducing histidine, phenylalanine, arginine, glycine, aspartic acid, serine, methionine valine, alanine, lysine, isoleucine, leucine, threonine, tryptophan, tyrosine, proline, citrulline, 4-hydroxyproline and gamma amino butyric acid (GABA). Metabolome results showed that fructose may aggravate experimental colitis symptoms by inducing amino metabolism dysbiosis in the colon. These findings suggested that fructose worsened colitis by manipulating the crosstalk between gut microbiota and their metabolites.

## 1. Introduction

Additive sweeteners such as sucrose and fructose are widely used in the food industry, and they have been reported to be associated with the increasing prevalence of metabolic syndromes and triggering tissue or organ function impairment. The liver plays a fundamental role in metabolism, such as secreting bile acids, storing or consuming glycogen and metabolizing toxins from foods [1]. A dynamic equilibrium is required for all organisms. Stress happens when homeostasis is broken, and it causes enormous influences and leads to non-communicable diseases (NCDs) [2]. Non-alcoholic fatty liver disease (NAFLD) is one of the major NCDs that is often diagnosed by the level of serum aspartate aminotransferase (AST), alanine aminotransferase (ALT), insulin resistance and hyperlipemia [3]. There are many research studies focused on revealing the mechanisms of sugar, especially fructose inducing obesity and other metabolic syndromes such as non-alcoholic fatty liver disease (NAFLD). The gut–liver axis is known as a bidirectional relationship between the gut, gut bacteria and liver. The gut and its bacterial health directly affect liver function [4]. It has been stated that fructose-inducing NAFLD is related to injuries relative to intestinal health by inhibiting the expressions of tight junction proteins (TJPs) and mucus proteins and enhancing the permeability of the gut. Excessive fructose intake primarily induced changes in gut permeability, leading to an increase in endotoxin levels in circulation and activated prototypical proinflammatory pathways such as toll-like receptors and the nuclear factor NF-kappaB (NF-κB), and this eventually results in NAFLD [5,6,7,8]. 

Rodent studies also provided direct evidence of the harmful effects of fructose in the colon. These studies stated that fructose decreased the thickness of colonic mucus and decreased epithelial barrier functions and increased endotoxin levels in plasma, which suggests that gut permeability increased. A high-fructose diet has been proven to alter the composition of gut microbiota in association with worsening dextran sodium sulfate (DSS)-induced colitis [9,10], while the mechanism of sweetener-induced gut impairments still needs to be clarified.

In recent years, the incidence rate of inflammatory bowel diseases (IBD) has increased worldwide for decades [11]. Dietary changes are generally believed to help stop the rising incidence of IBD. Although many factors have been implicated in IBD pathogenesis, sugar intake may be a potential and underappreciated contributor [10]. Another research suggested that dietary single sugars (glucose) alter gut bacteria and promote colitis in mice. It stated that the short-term overconsumption of sugar cannot alter gut bacteria in normal circumstances; however, sugar indeed exacerbated colon function in colitis mice, and this process has been proven to be involved in gut bacteria [10].

Since intestinal microbiota are important mediators of intestinal health [12,13], there is growing evidence supporting the observation that the gut microbiome–liver axis plays a crucial role in the pathogenesis of many metabolic diseases. Additive sugars are an energy source for both microbes and hosts, and they may alter bacterial nutrient sources and, at least in part, change the composition and population of certain microbes [14]. Then, bacteria influence the intestinal tract by regulating the levels and profiles of bile acids (BAs), short-chain fatty acids (SCFAs) and amino acids (AAs). The intestinal amino acids metabolism can mediate multiple immunity-related functions. Although the small intestine can transfer part of AAs into circulation, the large intestine contains more abundant microorganisms. Moreover, these microorganisms are widely involved in AA’s metabolism, producing various metabolites that are critical to hosts. Arginine mediates nitric oxide and regulates carbohydrate and lipid metabolism in the body [15], while proline metabolism mediates energy statuses and redox equilibrium from the cytosol to mitochondria [16]. Redox stress is known as a significant driver associated with multiple impairments and diseases [17]. In some circumstances, this dysbiosis in the gut could worsen metabolic and IBD syndromes [18].

The current study evaluated the influences of fructose on gut bacteria and their metabolites by applications in the microbiome and metabolome and using sucrose as a control. The potential mechanisms of additive sugar, especially fructose inducing colon inflammation via gut bacteria, are illustrated. We show that additive sugars worsen experimental colitis in rodent models and affect the microbiome, with changes in bacterial populations, compositions and their metabolites alterations. The role of amino acids (AAs) is suggested. Importantly, our findings stated a connection between fructose, microbial composition changes and their metabolic activity and intestinal inflammation.

## 2. Materials and Methods

### 2.1. Diets and Animal Experimental Design

Crystalline fructose (purity ≥ 99.9%) was obtained from XIWANG Food Co., LTD (Bingzhou, China); sucrose (purity ≥ 99.9%) was produced by KEAO XIELI Feed Co., LTD. (Beijing, China). The animal diets of each group are provided in Table S1. All diets contributed equally with respect to nutrients and caloric density.

All animal experiments complied with the ARRIVE guidelines, were carried out according to the National Research Council’s Guide for the Care and Use of Laboratory Animals and were approved by the Ethical Committee for Animal Experimentation of the Academy of National Food and Strategic Reserves Administration with utilization permission from Beijing Municipal Science & Technology Commission (No. SYXK(Jing) 2019-0015). In the experiment, 7-week-old male Sprague Dawley rats (specific-pathogen-free SPF grade, weighted 300–320 g) were purchased from Vital River Laboratory Animal Technology Co., Ltd. (Beijing, China). The rats were raised 3 per cage in an SPF laboratory room with feed and water ad libitum under 24 ± 2 °C; (60 ± 5)% relative humidity; and 12 h light/dark cycle environment conditions.

**Figure 1:**
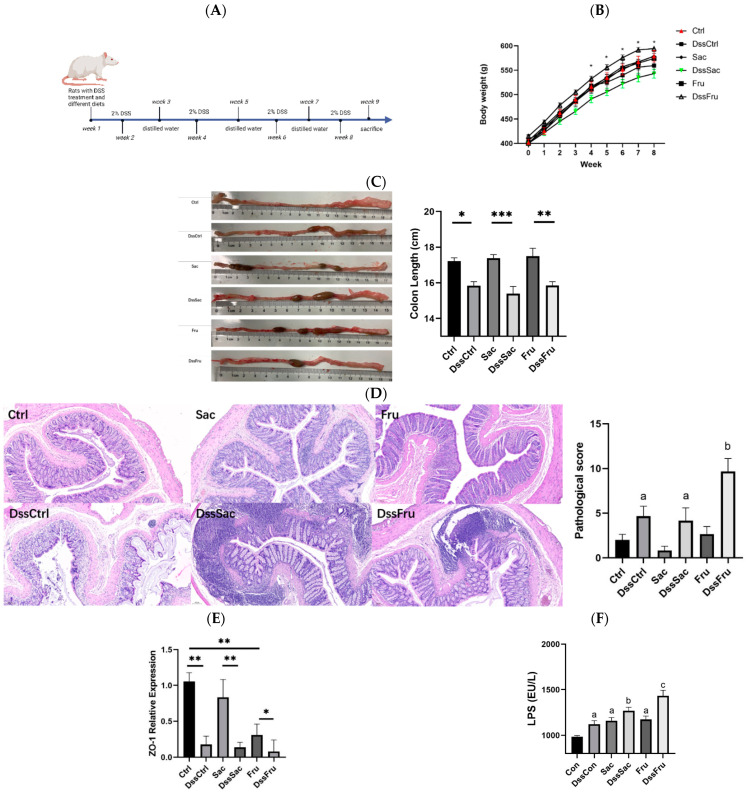
The addition of sugar, especially fructose, promoted the symptoms of DSS-induced colitis in rats by decreasing ZO-1 expression and increasing LPS levels. (**A**) Animal experimental design; (**B**) body weight of different groups (n = 10); (**C**) colon length appearance and statistical analysis (*n* = 6); (**D**) colon H&E staining (100×); (**E**) relative expression of ZO-1 (n = 6); (**F**) LPS level in serum, *n* = 8. Data represent the following: means ± SEM, * *p* ˂ 0.05, ** *p* ˂ 0.01, *** *p* ˂ 0.001. Different letters indicate a significant difference, *p* ˂ 0.05.

All rats were fed with AIN-93M for 1 week of adaption and then randomly divided into 6 groups (12 per group, 72 in total). The Ctrl group was fed sugar-free feeds, while Sac was fed 12.5% sucrose; Fru was fed 12.5% fructose in the diet (diet formula, Table S1). Ctrl, Sac and Fru were fed with distilled water. To induce colitis, DssCtrl, DssSac and DssFru groups were administered the same diet as Ctrl, Sac and Fru, respectively, and 2% DSS (Sigma-Aldrich, Shanghai, China) was provided in drinking water for 7 days and then altered to distilled water for 7 days for 4 cycles. The schematic overview of the animal experiment procedure is displayed in Figure 1A.

According to the recommendation of WHO (Organization, 2015), a 60 kg male adult (standard man) may not induce any health issues when intaking additive sugar amounting to no more than 50 g. Based on the equivalent dose conversion [19] of the average intake feed and body weight of rats, this additive sugar dose converted to rat doses was no more than 2.5 g. Therefore, the feed formula designed based on AIN-93M in our study contained approximately 12.75% of sucrose and fructose, and the DSS treatment groups applied the same feed formula correspondingly.

Food intake and body weight were measured weekly during the entire experimental period. All rats were sacrificed by cervical dislocation after brief carbon dioxide sedation. Subsequently, the colon’s length was measured, and colons containing feces and mucus were harvested and stored at −80 °C; blood samples were centrifuged to separate serum and then stored at −80 °C as well.

### 2.2. Histological Analysis 

The paraffin-embedded blocks of formalin-fixed individual colon sections were cut at 5 microns and stained with hematoxylin and eosin (H&E). Digital images were obtained by the DM2000 LED microscope (Leica Microsystems, Wetzlar, Germany). The criteria of pathological evaluation were based on the criteria of the degree of epithelial damage: 1 point = occasional mucosal ulcer formation; 2 points = 25–50% mucosal ulcer formation; 3 points = 51–75% mucosal ulcer formation; 4 points ≥ 75% mucosal ulcer formation; inflammatory cell infiltration degree; degree of crypt abscess; and reduction in goblet cells: 0 = no decrease; 1 point ≤ 10% reduction; 2 points = 10–25% decrease; 3 points = 25–50% reduction; 4 points = 50–100% reduction. Pathology scoring was performed in a blind manner by a pathologist at Peking University.

### 2.3. Oxidative Stress and Endotoxin Determination in Serum

Malondialdehyde (MDA) levels were measured by using commercially available biochemical assay kits following the manufacturer’s instructions (Nanjing Jiancheng Bioengineering Institute, Nanjing, China). Inflammation cytokine interleukin-6 (IL-6) and interleukin-8 (IL-8) were measured by using enzyme-linked immunosorbent assay ELISA kits (Nanjing Jiancheng Bioengineering Institute, Nanjing, China). Lipopolysaccharide (LPS) was determined by (ELISA) kits (Shanghai Enzymelinked Biotechnology Co., Ltd., Shanghai, China) according to the instructions of the kits. 

### 2.4. Tight Junction Protein Expression Determination 

All animal colon mucus was used to extract total RNA (Tiangen biochemical technology, Beijing, China), and RNA concentrations were determined by the NanoDrop system (ND5000, Bioteke, Wuxi, China). RNA was reverse transcribed to cDNA using the qScript cDNA Synthesis Kit (Tiangen biochemical technology, Beijing, China). Glyceraldehyde-3-phosphate dehydrogenase (Gapdh) was used as an endogenous normalization control for both designed and commercial primers. The amplified products of designed primers were verified by sequencing. qRT-PCR was performed using Fast SYBR Green Master Mix (Tiangen biochemical technology, China) on a real-time PCR system (CFX96, BIO-Rad, Hercules, CA, USA). Relative fold induction was determined using the ddCT (relative quantification) analysis protocol [20]. The primers were designed and purchased from Sangon Biotech (Sangon, Shanghai, China). 

### 2.5. Gut Microbacteria Analysis 

Bacterial DNA in colonic content samples was extracted using the OMEGA Soil DNA Kit (M5635-02) (Omega Bio-Tek, Norcross, GA, USA), following the manufacturer’s instructions, and stored at −20 °C prior to further analysis. The quantity and quality of extracted DNAs were measured using a NanoDrop NC2000 spectrophotometer (Thermo Fisher Scientific, Waltham, MA, USA) and agarose gel electrophoresis, respectively. The PCR amplification of the bacterial 16S rRNA gene’s V3–V4 region was performed using forward primer 338F (5′-ACTCCTACGGGAGGCAGCA-3′) and reverse primer 806R (5′-GGACTACHVGGGTWTCTAAT-3′). The PCR amplicons were purified with Vazyme VAHTSTM DNA Clean Beads (Vazyme, Nanjing, China) and quantified using the Quant-iT PicoGreen dsDNA Assay Kit (Invitrogen, Carlsbad, CA, USA). After the individual quantification step, amplicons were pooled in equal amounts, and pair-end 2 × 250 bp sequencing was performed using the Illumina NovaSeq platform with NovaSeq 6000 SP Reagent Kit (500 cycles) at Shanghai Personal Biotechnology Co., Ltd. (Shanghai, China). Microbiome bioinformatics was launched by QIIME2 2019.4

### 2.6. Metabolomics Analysis

Metabolomics analysis was performed by the Q300 Kit (Metabo-Profile, Shanghai, China). Ultraperformance liquid chromatography coupled to a tandem mass spectrometry (UPLC-MS/MS) system (ACQUITY UPLC-Xevo TQ-S, Waters Corp., Milford, MA, USA) was used to quantitate all targeted metabolites in this study by Metabo-Profile Biotechnology (Shanghai) metabolome manager temp.: 10 °C, mobile phases: A = water with 0.1% formic acid; B = acetonitrile/IPA (70:30); gradient conditions: 0–1 min (5% B), 1–11 min (5–78% B), 11–13.5 min (78–95% B), 13.5–14 min (95–100% B), 14–16 min (100% B), 16–16.1 min (100–5% B) and 16.1–18 min (5% B); flow rate: 0.40 mL/min; injection vol.: 5.0 µL. For the mass spectrometer, we have the following: capillary: 1.5 (ESI+), 2.0 (ESI−) Kv; source temp.: 150 °C; desolvation temp.: 550 °C; desolvation gas flow: 1000 L/h.

The raw data files were processed using the iMAP platform (v1.0; Metabo-Profile, Shanghai, China). Principal component analysis (PCA) and orthogonal partial least squares discriminant analysis (OPLS-DA) were also performed [21]. VIP (variable importance in projection) was obtained based on the OPLS-DA model. Metabolites with a VIP of ≥1 and *p*-value of < 0.05 (univariate analyses were based on whether the data were normally distributed) were regarded as statistically significant (differentially expressed metabolites: DEMs) [22]. The Z-score indicates how many standard deviations an observation is above or below relative to the mean of the control group. The V-plot that integrates the fold change and *p*-values is used for depicting significantly different metabolites.

## 3. Results

### 3.1. Fructose and Sucrose Enhanced DSS-Induced Phenotypes

To evaluate the effect of sugars on phenotypes, including the length, villi and crypt structure of the colon, SD rats with or without experimental colitis were subjected to sucrose and fructose feeds for 8 weeks (Figure 1A). The results show that the body weights of DssCtrl, DssSac and DssFru significantly decreased compared to their corresponding non-DSS treatment (Figure 1B, *p* < 0.05). The body weight of DssSac is lower than DssFru (*p* < 0.05). The colon length of DssCtrl, DssSac and DssFru was reduced compared to that of the non-DSS treatment (Figure 1C) (*p* < 0.05); however, the colon length between DSS treatment groups showed no statistical significance (*p* > 0.05). The pathological score is shown in Figure 1D. Within non-DSS treatments, modest sucrose and fructose did not induce pathological changes, while in the DSS treatment group, the pathological score of fructose was significantly higher than the control and sucrose group (*p* < 0.05), which suggested that fructose may worsen the impairment of colons in experimental colitis rats while sucrose not.

The level of intestinal TJ proteins, especially zonula occludens1 (ZO-1), is critically important with respect to the integrity and function of the gut barrier, and it was observed as significantly decreased in the colon of fructose-exposed rats in both with and without DSS treatment groups (Figure 1E, *p* < 0.05), while in sucrose-exposed rats, a decrease was only found in the DSS treatment group, which was similar to non-sugar-exposed rats. Lipopolysaccharides in the serum are regarded as endotoxins, and they increased in all sugar and DSS treatment groups; moreover, DssFru was the highest among all groups (Figure 1F, *p* < 0.05).

### 3.2. Fructose Induced Oxidative Stress and Inflammation in Serum

**Figure 2:**
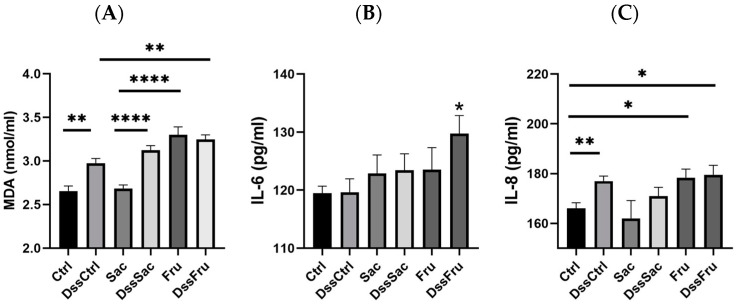
Fructose induced more oxidative stress and inflammatory levels in serum: (**A**) malondialdehyde (MDA) level in serum; (**B**) interleukin-6 (IL-6) level in serum; (**C**) interleukin-8 (IL-8) level in serum; n = 8. Data represent the following: means ± SEM, * *p* ˂ 0.05, ** *p* ˂ 0.01, **** *p* ˂ 0.0001.

Fructose intake resulted in a significant increase in serum MDA level in DSS and non-DSS treatment groups (Figure 2A, *p* ˂ 0.05), while the MDA level of the Sac group stayed at a similar level with Ctrl. Fructose also increased the IL-6 level significantly under DSS treatment conditions (DssFru) (Figure 2B, *p* ˂ 0.05). Furthermore, IL-8 levels increased in both Fru and DssFru groups (Figure 2C, *p* ˂ 0.05). Oxidative stress and inflammation responses are important clinical manifestations of metabolic syndromes. Our results show that compared to sucrose, fructose induced more severe oxidative stress injury even without DSS treatments.

### 3.3. Sucrose and Fructose Altered the Overall Structure and Composition of Gut Microbiota in Rats

**Figure 3:**
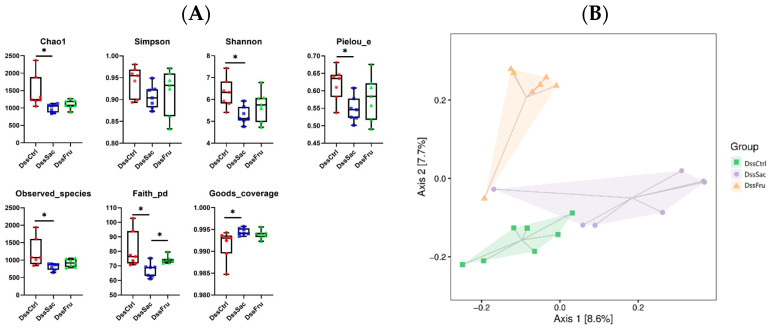
Effects of sucrose and fructose on the overall structure of gut bacteria within different conditions of rats. (**A**) α-diversity analysis; (**B**) PCoA score plot of colon contents based on Jaccards. n = 7. Data represent the following: means ± SEM, * *p* ˂ 0.05.

The rarefaction curve (Figure S1) approached the saturation plateau, indicating that OTUs were fully captured. A α-diversity analysis displayed that sucrose (DssSac) and fructose (DssFru) intake significantly reduced the gut microbial community’s richness compared to DssCtrl, as evidenced by the significantly decreased Chao1, Shannon, Pielou, observed species andfaith and goods coverage (Figure 3A, *p* ˂ 0.05). However, no significant difference in the gut microbial community’s richness was observed in non-Dss treatment groups (Figure S2A, *p* > 0.05). PCoA as a kind of β-diversity was employed to evaluate the sucrose and fructose intake on overall structural changes in the gut microbiota. The PCoA score plot of colon contents based on the Jaccard Index were applied. In the experimental colitis rodent model, PCoA displayed distinct clustering relative to the microbial community (Figure 3B, *p* ˂ 0.05) of three groups (DssCtrl, DssSac and DssFru), while the clustered microbial PCoA was similar without Dss treatment (Figure S2B, *p* > 0.05). The DssFru was evidently clustered, separating from the DssSac and DssCtrl, suggesting that fructose and sucrose may alter gut bacteria at different levels or even in an entirely different way.

**Figure 4:**
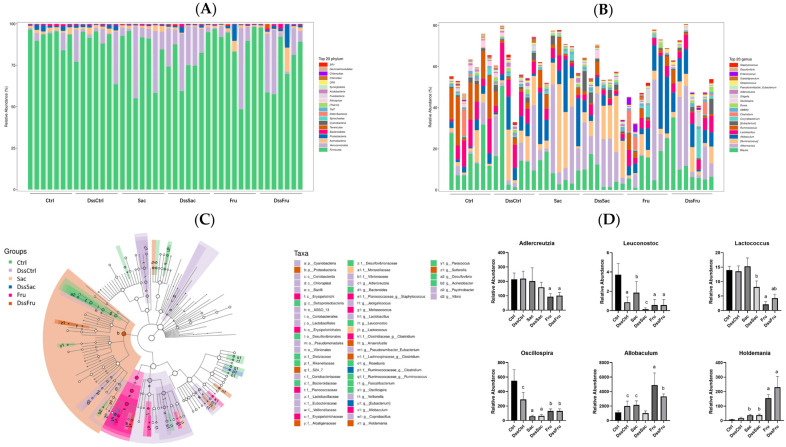
Alteration of sucrose and fructose on gut bacterial community in rats (*n* = 7). (**A**,**B**) Relative abundance of the fecal microbiota at the phylum and genus level. (**C**) Cladogram of different groups (LDA > 2). (**D**) Presentative differential taxa, *n* = 7. Data represent the following: means ± SEM; different letters indicate significant difference, *p* ˂ 0.05.

To further investigate the specific changes in gut bacteria composition caused by sucrose and fructose intake, two levels of taxonomic composition were analyzed, which were the phylum and genus. As shown in Figure 4A, the gut bacteria of rats were mainly Firmicutes, Verrucomicrobia, Actinobacteria and Proteobacteria, followed by Bacteroidetes. The taxonomic abundance presented significant reductions in the Bacteroidetes of all fructose intake groups, and this result seemed to have no relation with Dss treatments (Figure S3). However, no significant differences were observed in the Firmicutes/Bacteroidetes (F/B) ratio (Figure S3). LEfSe (linear discriminant analysis effect size) was performed to detect differentially abundant taxa across groups [13]. At the genus level, 24 OTUs have been identified as differential taxa (LDA ≥ 2, *p* < 0.0001), and differential taxa are listed in Figure 4D; the relative abundances of Adlercreutzia, Leuconoxtoc, Lactococcus, Oscillospira, Allobaculum and Holdemania are displayed in Figure 4D, while the rest of the differential taxa are shown in Figure S3. In our study, these three taxa were reduced significantly (LDA ≥ 2, *p* < 0.0001) in sucrose and fructose groups, and these reductions seemed unrelated to DSS treatments. Allobaculum [14] and Holdemania [15] were conditional pathogens, and they significantly increased (*p* < 0.05) in the fructose intake group. It is suggested that differential taxa were induced by fructose intake and not experimental colitis. In our previous study [16], Lachnospiraceas increased in the fructose intake group, and they increased again in this study (Figure S4). These results suggest that both sucrose and fructose could alter the relative abundance of certain taxa; however, fructose could induce more reductions in some probiotics and can increase the relative abundance of harmful or conditional pathogens to greater contents compared to sucrose.

### 3.4. Sucrose and Fructose Altered Colonic Content Metabolites Profiles in Experimental Rats

**Figure 5:**
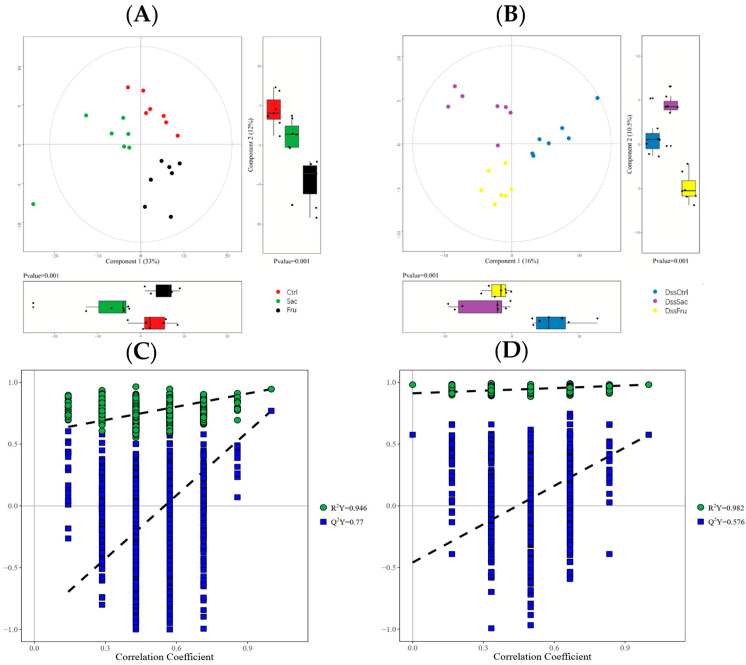
Metabolic profiles of sucrose and fructose in the colon contents of rats. (**A**,**B**) PCA score of colon contents in different groups; (**C**,**D**) OPLSA-DA permutation: C1. Sac vs. Fru; C2. DssSac vs. DssFru.

Gut bacteria are involved in the host’s metabolism as potential mechanisms that are strongly related to the metabolites of gut bacteria fermentation, especially in the lower digestive tract. To evaluate the metabolic profile alterations using sucrose and fructose with respect to their responses to gut bacteria changes, colonic content metabolites were analyzed by UPLC-MS/MS. A total of 218 metabolites were detected, including amino acids, bile acids, carbohydrates carnitines, fatty acids, imidazoles, organic acids, peptides phenylpropanoids and short-chain fatty acids (SCFAs). According to PCA plots (Figure 5A,B), a distinct clustering of colonic contents was observed in both normal and DSS treatment rat groups. Subsequently, the metabolic difference in colonic contents between sucrose and fructose was analyzed. OPLAS-DA was applied to evaluate data quality and screen biomarkers, and OPLAS-DA plots are shown in Figure S5. In the permutation test, interpretation parameter *R*^2^*Y* was nearly 1.0, the intercept of *Q*^2^*Y* was over 0.2, and the fitted curve on the Y-axis was <0, which hints at good reliability and the high predictability of the model (Figure 5C,D).

**Figure 6:**
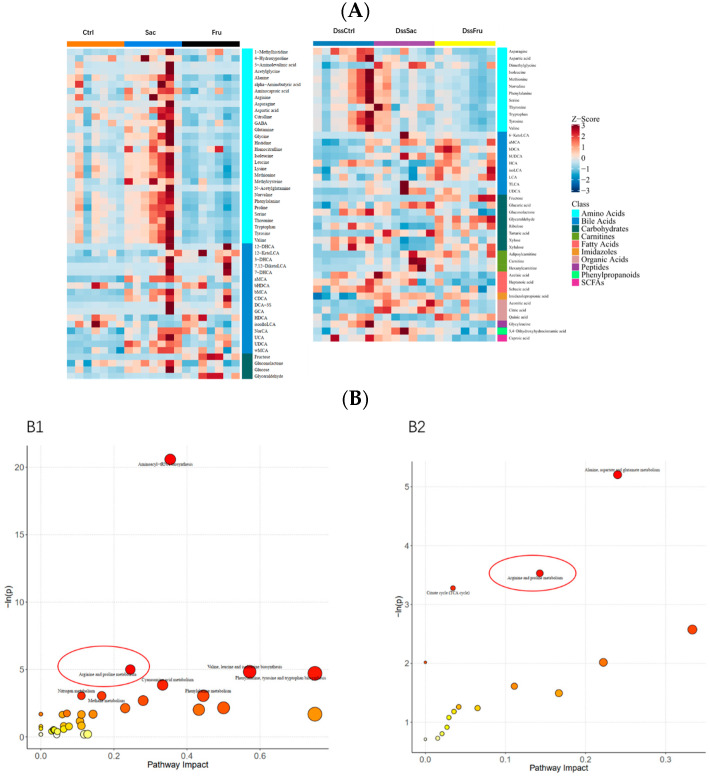
Sucrose and fructose alter colonic metabolite profiles. (**A**) Heatmap of sucrose and fructose’s alteration of metabolites; (**B**) KEGG annotation analysis of the altered metabolites based on the differences between sucrose and fructose; B1 KEGG annotation barplot of Sac vs. Fru; B2 KEGG annotation barplot of DssSac vs. DssFru. The arginine and proline metabolism pathways are highlighted with red circles in the figure.

Differential metabolites were identified based on the VIP values > 1.0 and *p* value < 0.05. The volcano plot (Figure S6) shows differential metabolites in different comparisons after 8 weeks of sucrose and fructose intake. In the non-DSS treatment group, fructose intake affected 73 upregulated and 5 downregulated metabolites compared to the sucrose group, while fructose altered 16 upregulated and 15 downregulated metabolites in the DSS treatment group. Specifically, the heatmap analysis (Figure 6A) exhibited major differential metabolites among each of the three groups (non-DSS treatment and DSS treatment), which mainly comprised amino acids, bile acids, carbohydrates, carnitines fatty acids, etc. Based on the differential metabolites between sucrose and fructose groups, a pathway enrichment analysis was launched. KEGG topology analyses were applied to evaluate metabolic changes induced by two different types of sugar (Figure 6B). Pathways were enriched in both non-DSS and DSS treatment groups, comparing sucrose and fructose intake and arginine and proline metabolism (Figure 6B). We next analyzed the pathways of arginine and proline metabolism, and this pathway is statistically significant (*p* < 0.05) in both non-DSS and DSS treatment groups, which means that the alterations of this pathway may be induced by different sugar intake and not experimental colitis. The enriched differential metabolites are listed in Table 1. 

Citrulline, aspartic acid, arginine, proline, 4-hydroxyproline and gamma amino butyric acid (GABA) are enriched in the arginine and proline metabolism pathway. Arginine and proline are related to immune system regulation and intracellular redox levels. Morreover, 4-hydroxyproline is often associated with protein degradation, and GABAs were proven to be beneficial neuroregulators. Nearly all differential metabolites were amino acids. This result suggested that fructose may induce the dysbiosis of amino acid metabolism in colons.

**Figure 7:**
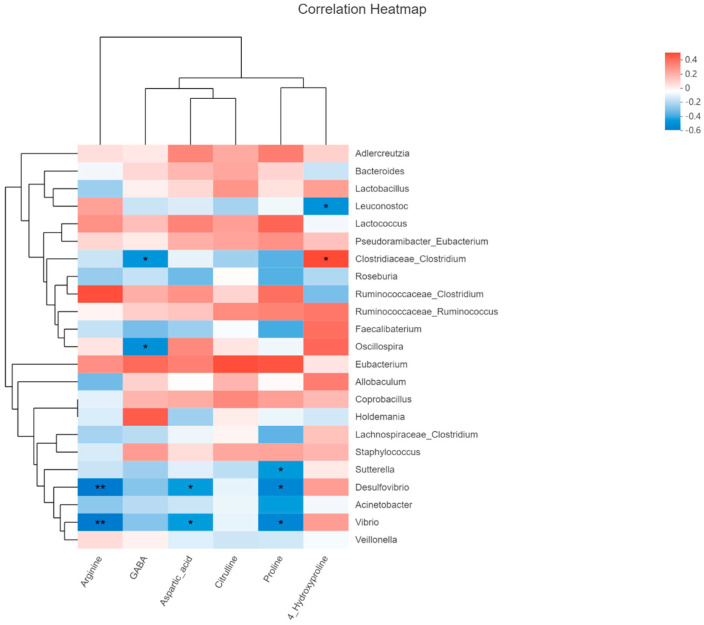
Heatmap of Spearman’s correlation between differential taxa and metabolites of rat, * *p* < 0.05, ** *p* < 0.01.

The pathway of arginine and proline metabolism (Table 1) has been selected to conduct Spearman’s correlation to analyze the relationship between differential taxa and metabolites (Figure 7) since this pathway has been enriched in both normal and DSS treatment rodent models. The level of arginine and aspartic acids was negatively related to Desulfovibio and Vibio; GABA is negatively related to Clostridium; proline had an inverse correlation with Desulfovibio, Vibio and Sutterella, while 4-Hydroxyproline was positively related to Clostridium and negatively related to Leuconostoc.

## 4. Discussion

Additive sugar is a kind of carbohydrate and also a source of energy to our body. The excessive intake of sugars leads to an imbalance in energy expenditure and finally induces metabolic disorders. Moreover, metabolic diseases have been stated as a major cause of death by the World Health Organization (WHO) [23,24]. Although IBD is not generally considered a metabolic disease, epidemiological research studies reported that the morbidity of IBD is associated with increasing the intake of carbohydrates [25,26]. Moreover, there are many research studies stating that fructose can increase intestine permeability, which leads to the accumulation of endotoxin in serum, subsequently inducing TLR4 in the liver and finally contributing to NAFLD [5,27]. However, the relationships between additive sugar and IBD are still debated since several clinical studies failed to discover any associations. Understanding the extrapolation of doses between species is important for pharmaceutical researchers when initiating new animal or human experiments. Interspecies allometric scaling for dose conversion from animal to human studies is one of the most controversial areas in clinical pharmacology [24,25,26]. Although some research studies indeed suggested that fructose can decrease the thickness of mucus in colons and induce inflammations in the bowel, these mechanisms require further explanations. Therefore, our current study provided evidence of the potential mechanisms affected by fructose intake in exacerbating IBD.

The dose of no more than 2.5 g of sugar in this study is not considered a high amount for sugar intake. In normal conditions (without DSS treatment), sugar intake did not induce significant changes in colonic pathology, while both sucrose and fructose were found to promote the infiltration of inflammation under DSS treatment conditions based on colon pathology and inflammatory factors. Moreover, fructose produced a more serious effect (Figure 1 and Figure 2). These results proved that sugar, especially fructose intake, can induce inflammation in the colon and worsen symptoms under colitis conditions. To clarify the mechanisms, the regulated effects of fructose and sucrose on colonic flora were mainly investigated and compared in this research study. We listed all the differential flora and their potential functions in the gut (Table 2).

The relative abundance of differential taxa is shown in Figure 4D and Figure S4. Probiotics including *Adlercreutzia*, *Leuconostoc*, *Lactococcus* and *Oscillospira* were significantly reduced in the fructose intake group, while some bacteria associated with induced inflammation and diseases (*Allobaculum* and *Holdemania*, Figure 4D) were observed to be enriched in fructose intake group. *Adlercreutzia* [27], *Leuconoxtoc* [31], *Lactococcus* [32] and *Oscillospira* [42] were proven as probiotic or had a positive correlation with human/animal health. It is hinted that even though both sucrose and fructose changed gut bacteria in experimental colitis rats, fructose levels tended to change for the worse situation. Differential metabolites and regulated pathways are shown in Table 1. Arginine and proline metabolism were altered in normal and DSS treatment rats, which suggested that this alteration was related to different additive sugar intake and not experimental colitis. In this case, citrulline, aspartic acid, arginine, proline, 4-hydroxyproline and GABA can be regarded as the biomarkers of fructose that induce the impairment of colon health. According to the correlation analysis, future research studies could focus on the validation of the relationships between differential taxa and metabolites.

Our research study found that fructose-induced metabolism imbalance in many amino acids. Amino acids’ metabolism is fundamental not only with respect to protein constitution, but it is also fundamental for controlling immune cell function, regulating T cell fate, supporting metabolic rewriting and promoting glycolysis and mitochondrial metabolism. Furthermore, amino acids control sulfur and redox metabolism; as a result, the accumulation of endotoxin, ROS and amino acid dysbiosis occurs [51]. Combined with data in this paper, it can be suggested that the structural alteration of gut bacteria would induce the accumulation of endotoxin, further lead to amino acids dysbiosis and finally the deterioration of the colonic microenvironment. Similar results were also found, and they show that high fructose diets reprogram glutamine-dependent oxidative metabolism to enhance inflammation [52].

The aim of this study was to screen the potential changes induced by high fructose intake. The innovation of this experimental design was to compare the changes in normal and colitis models. The common changes in different models produced reliable microbiome and metabolomics results. From our multi-omics results, both sucrose and fructose could induce potential colonic inflammation, and fructose has a stronger effect when promoting colitis by using endotoxins and increasing MDA in the serum and decreasing ZO-1 expression; meanwhile, it altered the composition and structure of gut bacteria by reducing probiotics ranging from Adlercreutzia, Lactobacillus, Roseburia, Leuconostoc and Lactococcus to Oscillospira and increasing taxa that are associated with colitis, such as Allobaculum, Coprobacillus, Holdemania, etc. Moreover, it changed the metabolic profile of colonic contents by reducing the level of citrulline, aspartic acid, arginine, proline, 4-hydroxyproline and GABA and finally inducing amino metabolism dysbiosis. For future research studies, the results need to be validated using in vivo and in vitro experiments. Our results suggest that the dietary recommendations for IBD patients, especially with respect to additive sugar, need applied with caution. As avoiding dietary fiber is a main dietary advice for colitis patients, additive sugar restrictions need to be considered.

## Figures and Tables

**Table 1:** Effects on fructose intake compared to sucrose in non-DSS treatment.

No.	Pathway	Hits	Raw P	Impact	Enriched Compounds
1	Aminoacyl-tRNA biosynthesis	16	1.15 × 10^−9^	0.35412	Histidine (↓) Phenylalanine (↓) Arginine (↓) Glycine (↓) Aspartic acid (↓) Serine (↓) Methionine (↓) Valine (↓) Alanine (↓) Lysine (↓) Isoleucine (↓) Leucine (↓) Threonine (↓) Tryptophan (↓) Tyrosine (↓) Proline (↓)
2	Arginine and proline metabolism	6	0.006732	0.24491	Citrulline Aspartic acid (↓) Arginine (↓) Proline (↓) 4-Hydroxyproline (↓) Gamma amino butyric acid (GABA) (↓)
3	Valine, leucine and isoleucine biosynthesis	3	0.007934	0.57143	Leucine (↓) Valine (↓) Isoleucine (↓)
4	Phenylalanine, tyrosine and tryptophan biosynthesis	2	0.008931	0.75	Phenylalanine (↓) Tyrosine (↓)
5	Cyanoamino acid metabolism	2	0.021203	0.33333	Glycine (↓) Serine (↓)
6	Nitrogen metabolism	2	0.047114	0.11111	Histidine (↓) Glycine (↓)
7	Methane metabolism	2	0.047114	0.16667	Glycine (↓) Serine (↓)
8	Phenylalanine metabolism	2	0.047114	0.44444	Phenylalanine (↓) Tyrosine (↓)

**Table 2:** Differential taxa and potential biological functions in the gut.

		Potential Biological Effect	Potential Function	References
1	Adlercreutzia	Beneficial	Ulcerative colitis biomarkers	[27]
2	Bacteroides	Beneficial/harmful	Anti-inflammatory/pro-inflammatory	[28,29]
3	Melissococcus	NA		
4	Lactobacillus	Beneficial	Improve immunity	[30]
5	Leuconostoc	Beneficial	Improve immunity	[31]
6	Lactococcus	Beneficial	Improve immunity	[32]
7	Anaerofustis	NA		
8	(Pseudoramibacter) Eubacterium	Harmful	Pro-inflammatory	[33,34]
9	(Clostridiaceae) Clostridium	Harmful	Pro-inflammatory Infectious	[35,36]
10	Roseburia	Beneficial	Anti-inflammatory Improve immunity	[37]
11	(Ruminococcaceae) Clostridium	Harmful	Pro-inflammatory Infectious	[38]
12	(Ruminococcaceae) Ruminococcus	Beneficial	Anti-inflammatory/pro-inflammatory	[34,39,40]
13	Faecalibaterium	Beneficial	Fatty-acid-producing bacteria	[41]
14	Oscillospira	Beneficial	Butyrate-producing-bacteria	[42]
15	(Erysipelotrichaceae) Eubacterium	Harmful	Pro-inflammatory	[33,34]
16	Allobaculum	Harmful	Induce colitis	[43]
17	Coprobacillus	Harmful	Proinflammatory	[39]
18	Holdemania	Harmful	Anxiety	[41]
19	Paracoccus	NA		
20	(Lachnospiraceae) Clostridium	Harmful	Pro-inflammatory Infectious	[33,39]
21	Staphylococcus	Harmful	Infectious	[44]
22	Jeotgalicoccus	NA		
23	Sutterella	Pathogen	Diarrhea	[45]
24	Desulfovibrio	Pathogen	H2S producing	[46]
25	Acinetobacter	Pathogen	Infectious	[47]
26	Psychrobacter	NA		[48]
27	Vibrio	Pathogen	Infectious	[49]
28	Veillonella	Beneficial	Inhibit toxic bile acids	[50]

## Data Availability

Not applicable.

## Supplementary Materials

The following supporting information can be downloaded at https://www.mdpi.com/article/10.3390/nu15030782/s1. Figure S1: The rarefaction curve and Shannon index curve. Figure S2. Gut microbiota divergence of rats in the addition of sucrose and fructose under non-DSS treatment conditions. (A) Overall structure of gut bacteria; (B) PCoA score plot of colon contents based on Jaccards; the taxonomic abundance of *Bacteroidetes* and the ratio of *Firmicutes/Bacteroidetes*, * *p* < 0.05, n = 7; Figure S4. Relative abundance of OTUs of which the LDA score is ≥ 2; different letters present significant differences, *p* < 0.05, *n* = 7. Figure S5. Score plots of OPLS-DA of different groups. Figure S6. Volcano plots of colonic metabolites of rats showing significantly changed metabolites in groups: A. Sac vs. Fru; B. DssSac vs. DssFru. Table S1: The feed formula.
