# Supplementary material for: Fructose Stimulated Colonic Arginine and Proline Metabolism Dysbiosis, Altered Microbiota and Aggravated Intestinal Barrier Dysfunction in DSS-Induced Colitis Rats
Source: Nutrients. 2023 Feb 3;15(3):782. doi: 10.3390/nu15030782 (PMC9921751; doi:10.3390/nu15030782)
Supplement: Supplementary file 1 [file nutrients-15-00782-s001.zip › nutrients-2178651-supplementary.pdf]

A Supplementary materials

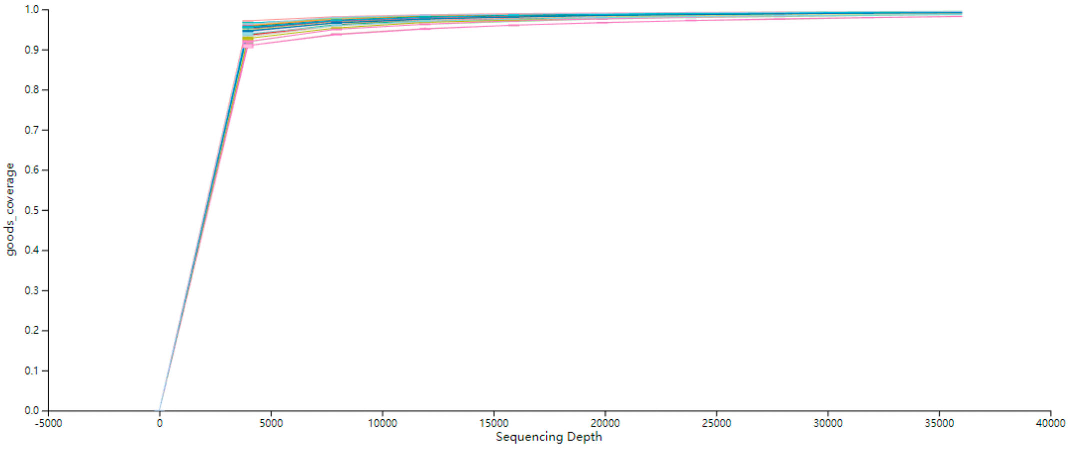

Figure.S1 The rarefaction curve and Shannon index curve

A

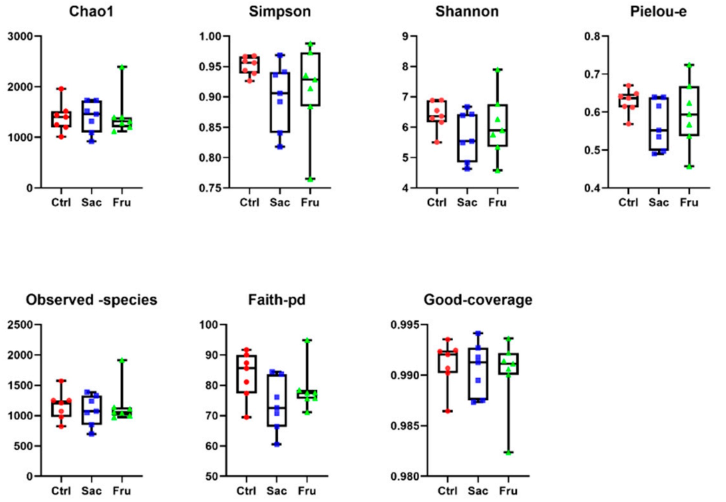

B

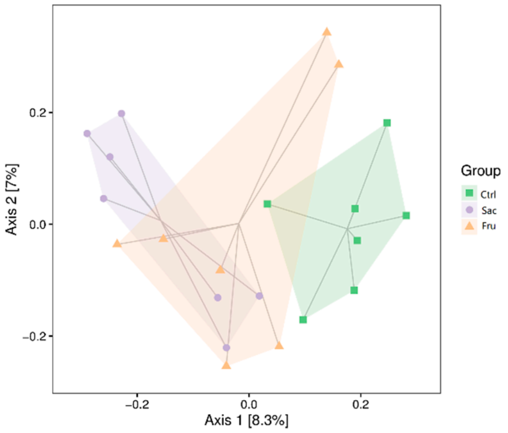

Figure.S2 Gut microbiota divergence of rats in addition of sucrose and fructose under non-DSS treatment conditions (A) Overall structure of gut bacteria; (B) PCoA score plot of colon contents base on jaccards.

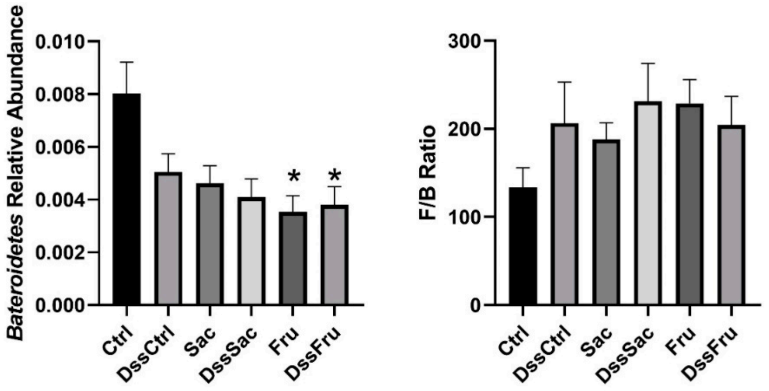

Figure S3 The taxonomic abundance of *Bacteroidetes* and the ratio of *Firmicutes/ Bacteroidetes*， *$P < 0.05$, n=7.

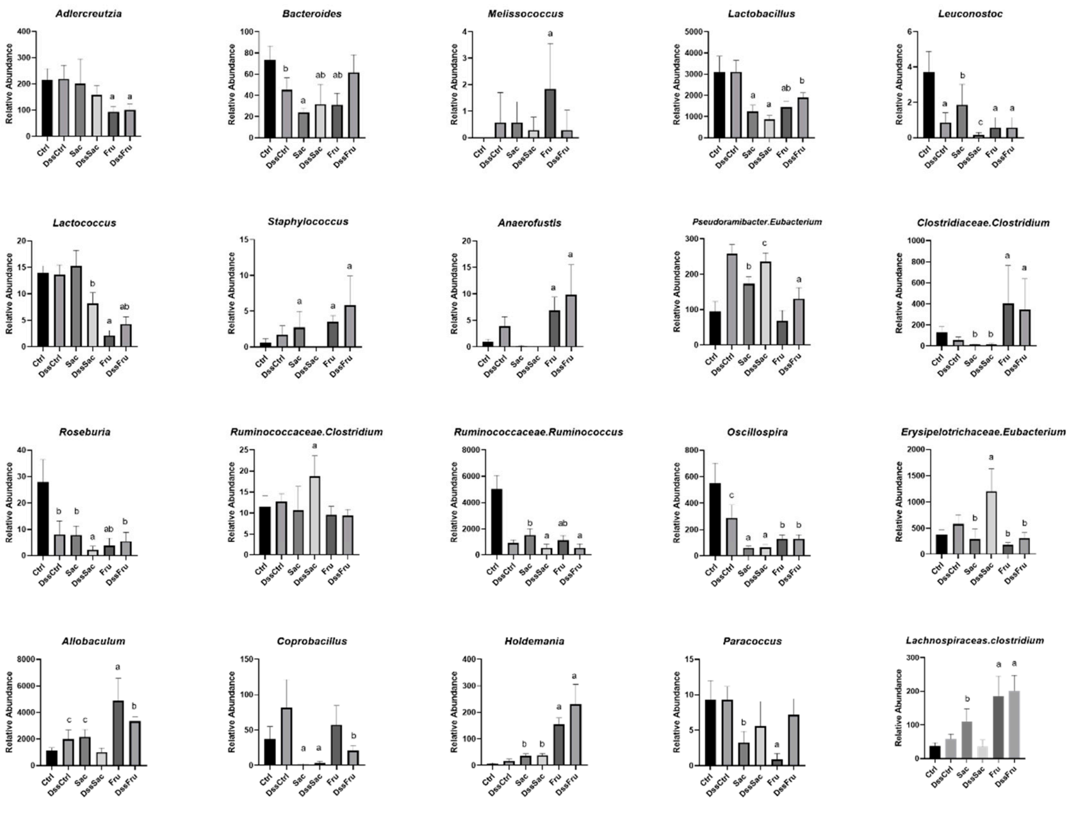

Figure.S4 Relative abundance of OTUs of which LDA score≥2, different letters present significant differences, $P < 0.05$, n=7

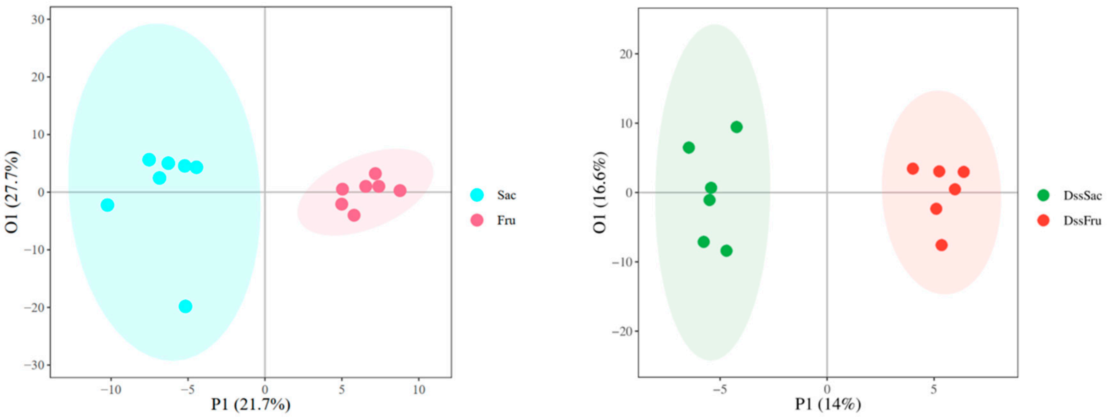

Figure.S5 Scores plots of OPLS-DA of different groups

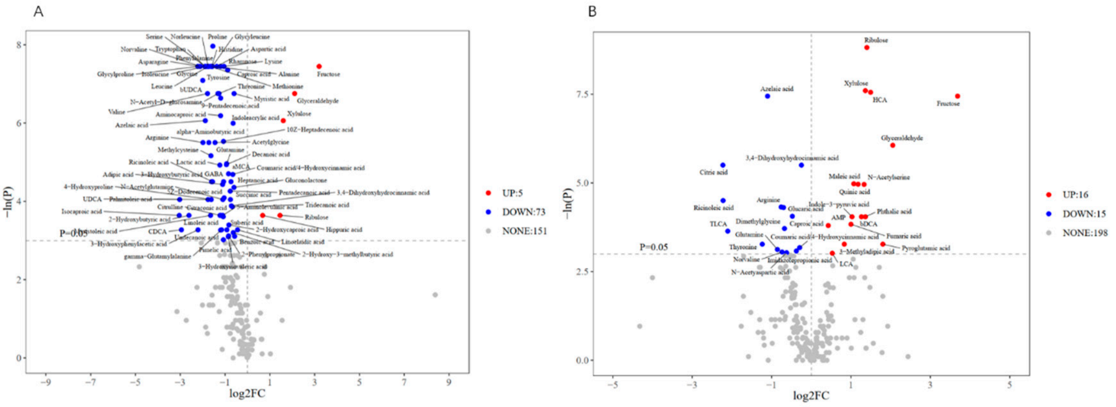

Figure.S6 Volcano plots of colonic metabolites of rats showing the significantly changed metabolites in groups, A. Sac vs Fru, B. DssSac vs DssFru

Table S1. The feed formula

	Con		Sac		MFD	
	g%	kcal%	g%	kcal%	g%	kcal%
protein	19.24	19.24	19.24	19.24	19.24	19.24
Carbohydrates	71.09	66.35	71.09	66.35	71.09	66.35
fat	4.27	14.41	4.27	14.41	4.27	14.41
casein	18.96	75.84	18.96	75.84	18.96	75.84
l-cystine	0.28	1.12	0.28	1.12	0.28	1.12
Corn starch	66.35	265.4	53.6	214.4	53.6	214.4
fructose	0	0	0	0	12.75	51
sucrose	0	0	12.75	51	0	0
cellulose	4.74	0	0	0	4.74	0
Soybean oil	2.37	21.33	2.37	21.33	2.37	21.33
lard	1.9	17.1	1.9	17.1	1.9	17.1
Multiple mine	0.95	0	0.95	0	0.95	0
Calcium hydrogen phosphate	1.23	0	1.23	0	1.23	0
Calcium carbonate	0.52	0	0.52	0	0.52	0
Potassium citrate	1.56	0	1.56	0	1.56	0
multidimensional	0.95	0	0.95	0	0.95	0
Choline bitartrate	0.19	0	0.19	0	0.19	0
In total	100	380.79	100	380.79	100	380.79
